# Pancreatic stone protein as a biomarker of sepsis

**DOI:** 10.1186/s13054-022-03953-x

**Published:** 2022-04-08

**Authors:** Diogo Lopes, Beatriz Chumbinho, João Pedro Bandovas, Pedro Faria, Catarina Espírito Santo, Bernardo Ferreira, Luis Val-Flores, Rui Pereira, Nuno Germano, Luís Bento

**Affiliations:** 1 Department of Intensive Care Medicine (Unidade de Cuidados Intensivos Polivalente), Curry Cabral Hospital, Central Lisbon University Hospital, Lisbon, Portugal; 2 Department of General Surgery, Curry Cabral Hospital, Central Lisbon University Hospital, Lisbon, Portugal; 3 Department of Intensive Care Medicine (Unidade de Urgência Médica), São José Hospital, Central Lisbon University Hospital, Lisbon, Portugal; 4 NOVA Medical School, Universidade Nova de Lisboa, Lisboa, Portugal

Dear Editor,

Regarding the extensive review published in *Critical Care* on biomarkers for sepsis by Dr. Barichello and co-workers [1], we would like to add a comment concerning an absent biomarker. *Do biomarkers in patients with sepsis or septic shock predict mortality, MODS, or organ dysfunction?* was the question addressed and after analysing around sixty biomarkers, they concluded that *significant work is needed to identify the optimal combinations of biomarkers that can augment diagnosis, treatment, and good patient outcomes*. Surprisingly, they do not mention a quite studied biomarker: the Pancreatic Stone Protein (PSP).

PSP is a 16 kDs C-type lectin protein produced mostly by the pancreas and the intestine. Originally described in 1990 as a protein secreted by pancreatic acinar cells to inhibit growth and nucleation of calcium carbonate crystals in the pancreatic juice, subsequent preclinical studies have shown that PSP is actually a damage-associated molecular patterns (DAMPs) produced as a response to sepsis.

PSP has been the subject of 13 clinical studies with 1674 unhealthy patients until 2019. Overall, comparing to routine biomarkers, namely C-reactive protein (CRP) and procalcitonin (PCT), PSP has shown to be more specific and sensitive on infection and sepsis diagnosis in different clinical settings (adults, pediatrics, post-operative, trauma, emergency and intensive care departments).

In 2020, a prospective study in 90 severely burned patients showed that PSP rises significantly earlier (up to 72 h) than the onset of clinical signs of sepsis [2]. PSP accuracy was superior to PCT on sepsis prediction with an area under the curve (AUC) in receiver operating characteristic (ROC) analysis of 0.89—95%CI 0.81–0.97 vs. 0.86—95% CI 0.77–0.94, respectively, and even higher (0.90 to 0.92) if both combined. Early identification of sepsis was confirmed by a prospective multicenter study with 243 critically ill patients from 14 centers that was published in 2021 in *Critical Care* [3], where PSP preceded clinical diagnosis even earlier (5 days), comparing to PCT and CRP (3 and 2 days, respectively). A contemporary meta-analysis (5 studies and 631 patients) [4] concluded that PSP is “a promising biomarker to diagnose infections in hospitalized patients”, outperforming CRP or PCT on discriminating infection from non-infection.

Furthermore, on patients admitted for sepsis, the value of PSP at admission correlates with SOFA score (severity of sepsis) and predicts ICU mortality. In COVID-19 patients PSP revealed a potential role as triage tool in emergency department due to its excellent negative predictive value for 7-day mortality [5].

Lastly, knowing that early recognition of sepsis is essential and a major determinant of the outcome, point-of-care testing as the one available for PSP is very appealing, as it provides results at bedside in less than 10 min.

For all these reasons, as in previous reviews, we consider PSP must be included in any manuscript regarding sepsis diagnosis.

## Data Availability

Not applicable.
