# Supplementary material for: Pancreatic stone protein as a biomarker of sepsis
Source: Crit Care. 2022 Apr 8;26:100. doi: 10.1186/s13054-022-03953-x (PMC8990498; doi:10.1186/s13054-022-03953-x)
Supplement: Related texts

# Reply: Is pancreatic stone protein (PSP) a biomarker for sepsis?

**Authors:** Tatiana Barichello, Mervyn Singer, Felipe Dal-Pizzol

**Affiliations:** 5 grid.267308.8 0000 0000 9206 2401 Faillace Department of Psychiatry and Behavioral Sciences, McGovern Medical School, The University of Texas Health Science Center at Houston (UTHealth), 1941 East Road, Suite 3140, Houston, TX 77054 USA; 6 grid.412287.a 0000 0001 2150 7271 Laboratory of Experimental Pathophysiology, Graduate Program in Health Sciences, University of Southern Santa Catarina (UNESC), Criciúma, SC Brazil; 7 grid.83440.3b 0000000121901201 Bloomsbury Institute of Intensive Care Medicine, University College London, London, UK

We thank Dr. Lopes and colleagues for their letter and for providing new publications that post-dated our literature search [1]. We do readily acknowledge that pancreatic stone protein (PSP), among others, could also have been included as biomarkers of interest in our narrative review [1] but such a review is, by definition, not intended to be all-inclusive. We are uncertain as to the underlying mechanism by which serum PSP levels rise in septic patients. In Fig. 1, we offer some speculative thoughts, suggesting that sepsis triggers an imbalance in the host's immune response mechanisms triggering cell or organ dysfunction and a resulting increase in serum PSP/reg 1 levels. We would also highlight issues relating to specificity as Dr. Lopes and colleagues did not mention that PSP also rises in acute and chronic pancreatitis, pancreatic and gastric cancers, pediatric acute osteomyelitis, type 2 diabetes mellitus, chronic renal failure [6], and in postoperative patients with secondary peritonitis admitted to the intensive care unit (ICU) [7]. Notably, the recent systematic review by Prazak et al. [4] suggests a PSP cut-off value of 44.2 ng/ml in patients diagnosed with an infection in the emergency department or ICU, while Pugin et al. [3] identified a cut-off value of 290.5 ng/ml in patients developing sepsis while in the ICU with non-septic patients having levels exceeding 150 ng/ml throughout their ICU stay. Clearly, a better understanding of what a PSP level represents in specific patient populations is needed. Crucially, like every proposed sepsis biomarker, PSP must also pass the acid test of being able to direct definitively change patient management and improve outcomes.

**Fig. 1:** PSP/reg1 and sepsis. **A** Distribution of PSP/reg1 in a healthy human digestive system and the deposition in the brain of Alzheimer's disease patients. **B** Hypothetical model of PSP's involvement in sepsis. Sepsis triggers an imbalance of the host immune response triggering cell or organ dysfunction, increasing the PSP/reg 1 in the serum of the septic patients. Amyloid-beta (Aβ), neurofibrillary tangles (NFL), pancreatic stone protein (PSP)
